# Metabolic psychiatry and the epistemic risk of somatic proxies

**DOI:** 10.1016/j.nsa.2026.107031

**Published:** 2026-10-05

**Authors:** Stefan Jerotic

**Affiliations:** a Clinic for Psychiatry, University Clinical Centre of Serbia, Belgrade, Serbia; b Faculty of Medicine, University of Belgrade, Belgrade, Serbia

The two studies published in *Neuroscience Applied* contribute to a rapidly consolidating literature indicating that psychiatric and metabolic traits overlap across several levels of analysis. Bouzouina et al. (2026) report, in a large bi-centric cohort comprising patients with major depressive disorder and healthy controls, that body mass index (BMI) is associated with a widespread subnetwork of the structural brain connectome. Higher BMI was associated with greater tractography-derived streamline counts within this network, and no statistically significant BMI-by-diagnosis interaction was detected. Although BMI was also associated with depressive symptom severity and anhedonia, connectivity within the BMI-associated network was not associated with depressive symptom severity and was not independently associated with anhedonia once BMI itself was taken into account. Šakić et al. (2026), using genomic structural equation modelling, similarly move beyond conventional diagnostic case–control comparisons. They identify one latent genetic factor on which attention-deficit/hyperactivity disorder symptoms, BMI, fasting plasma glucose, and glycated haemoglobin loaded, and another on which a combined obsessive-compulsive symptom/OCD phenotype and glycated haemoglobin loaded. These results demonstrate genetic covariance among the included traits and extend the observation beyond diagnostic case–control contrasts; they do not identify a causal pathway or establish that insulin signalling mediates the overlap.

Taken together, the studies are consistent with a transdiagnostic and dimensional model of metabolic–psychiatric overlap and with, consistent with broader evidence that depression, neurodevelopmental traits, insulin signalling, and obesity may share biological pathways (Fanelli et al., 2022; Milaneschi et al., 2019). Their importance lies precisely in their resistance to simple reductionism. Bouzouina et al. (2026) do not demonstrate that BMI-related connectomic alterations constitute a neural mechanism of depression or anhedonia; rather, they identify a cross-sectional association between BMI and a tractography-derived cortical network measure across diagnostic groups. Likewise, Šakić et al. (2026) do not show that insulin resistance causes ADHD or OCD. They identify shared genetic architecture among dimensional psychiatric and insulin-related traits. The conceptual implication is therefore subtler than metabolic reductionism: psychiatric vulnerability, metabolic regulation, reward processing, neurodevelopment, and brain-network organisation may may overlap across conventional diagnostic categories, but the biological processes, directionality, and clinical relevance of that overlap remain unresolved.

Four levels of inference should therefore be kept separate. Statistical association establishes covariation. Shared genetic architecture indicates partly overlapping inherited influences but does not, by itself, establish a shared biological pathway. Mechanistic understanding requires designs capable of testing directionality and mediating processes. Biomarker utility, in turn, requires reproducible prospective evidence that a measure provides clinically useful information about risk, diagnosis, prognosis, or treatment response. Neither focal study evaluates predictive performance, disease progression, treatment response, clinical stratification, or patient benefit. Their findings are therefore best understood as association-level and hypothesis-generating, rather than as validation of clinically actionable biomarkers. More specifically, the cross-sectional design of Bouzouina et al. precludes temporal or causal inference. BMI is a limited surrogate that does not distinguish adiposity, fat distribution, body composition, or insulin sensitivity. Although the analyses accounted for age, sex, diagnosis, and scanner site, residual confounding by physical activity, diet, socioeconomic circumstances, medication exposure, and somatic comorbidity remains possible. Moreover, tractography-derived streamline counts are an indirect measure of structural connectivity, and the cortical-only parcellation omitted subcortical reward circuitry. The absence of an independent relationship between the identified network and depressive symptoms or anhedonia further limits its interpretation as directly relevant to depression pathophysiology. In Šakić et al., genomic structural equation modelling summarises genetic covariance but cannot establish causal direction or identify the biological processes producing it; the latent factors may reflect broad pleiotropy rather than an insulin-specific pathway. The contributing GWAS datasets also differed in age, phenotype definition, and sample size, included symptom proxies that are not equivalent to clinical diagnoses, and were restricted to populations of European ancestry. No individual SNP reached genome-wide significance after heterogeneity filtering, while the gene-level, pathway, and tissue-expression findings remain hypothesis-generating rather than mechanistic validation. Translation of either study into clinical interventions or stratification is therefore currently speculative.

This increasing biological sophistication nevertheless creates an important epistemic risk. Once metabolism becomes psychiatrically meaningful, bodily measurements may begin to operate as psychiatric proxies. BMI, glycaemic indices, body shape, and weight change may be interpreted too readily as evidence of mental state, prognosis, self-regulation, or treatment success. BMI is particularly vulnerable to this overextension because it is both an imprecise indicator of individual metabolic health and a socially saturated sign (Tomiyama et al., 2016). Body weight continues to carry moralised associations with discipline, willpower, responsibility, and personal worth, while higher-weight individuals are frequently stereotyped as lacking self-control (Nutter et al., 2016; Puhl and Heuer, 2010). Weight stigma is itself associated with poorer mental health, including depression, anxiety, psychological distress, and disordered eating (Emmer et al., 2020), and evidence indicates that body size can influence mental-health professionals’ clinical judgements and treatment recommendations (Philip et al., 2025). The emerging science of metabolic psychiatry could therefore unintentionally intensify this symbolic economy if findings about associations are translated into the simplified proposition that a heavier body is a more depressed, poorly regulated, or psychiatrically vulnerable body.

Hacking's account of looping effects provides a useful framework for understanding this danger. In his original formulation, classifications of human beings can alter the self-understanding and behaviour of those classified, whose responses may subsequently modify the classifications themselves (Hacking, 1995, 2007). Gauld et al. (2025) further conceptualise looping as an epistemic process mediated by patients, clinicians, expert communities, and diagnostic systems. They introduce the concept of “looping breaks” to describe situations in which patient testimony and experiential knowledge fail to influence clinical or nosological understanding because of testimonial or hermeneutical injustice. The two metabolic studies invite consideration of an adjacent phenomenon: the somatic proxy loop.

The somatic proxy loop denotes a related process. It begins when a bodily measurement is interpreted as evidence of psychiatric state. That interpretation may shape clinical decisions and self-understanding; the ensuing behavioural, social, or treatment-related changes may then affect both the bodily measure and the person's experience; and those changes may appear to confirm the original interpretation. Unlike Hacking's original loop, its initiating object is a somatic metric rather than a classification of persons. Unlike a looping break, it describes a feedback process rather than a failure of experiential knowledge to influence classification, although it may produce such a break when bodily data displace the person's testimony.

This point is not a criticism of the empirical studies themselves. On the contrary, both papers are careful: Bouzouina et al. explicitly show that BMI-related connectivity did not independently account for symptom severity, and Šakić et al. emphasise shared liability rather than deterministic causation. The concern lies in translation. As metabolic psychiatry enters clinical practice, body metrics – and any future biomarkers that undergo separate validation – may acquire excess interpretive authority. This can create a form of epistemic shortcut: visible or measurable bodily change becomes a stand-in for psychiatric understanding. The expanding use of GLP-1 receptor agonists provides a forward-looking example. Marked weight loss might be interpreted socially or clinically as psychiatric improvement even when recovery has not been independently assessed. Whether such misattribution occurs, and with what consequences, remains an empirical question; the example illustrates a possible translational risk rather than supporting an inference about the psychiatric effects of GLP-1 receptor agonists.

On this basis, contemporary psychiatry requires a double movement. First, psychiatry should take metabolic biology seriously, because accumulating evidence indicates meaningful overlap across processes conventionally divided into “mental” and “somatic” domains. Second, psychiatry must resist the moral and epistemic overuse of metabolic markers. BMI and insulin-related traits should be treated as potentially relevant dimensions whose interpretation should be limited to the clinical questions for which they have been validated, not as proxies for personhood, agency, or mental wellness. The promise of metabolic psychiatry lies not in replacing psychiatric categories with somatic ones, but in developing a more careful account of how biological vulnerability, lived embodiment, and social interpretation interact.

## Conflicts of interest

None.
